# Influence of Laser Treatment of Ti6Al4V on the Behavior of Biological Cells

**DOI:** 10.3390/ma17092008

**Published:** 2024-04-25

**Authors:** Simon Syrovatka, Pavel Kozmin, Frantisek Holesovsky, Martin Sorm

**Affiliations:** 1 Department of Machining Technology, Faculty of Mechanical Engineering, University of West Bohemia, 30100 Pilsen, Czech Republic; holesovf@fst.zcu.cz; 2 HOFMEISTER s.r.o., 30100 Pilsen, Czech Republic; kozmin@hofmeister.cz; 3 Department of Materials and Technology, Faculty of Electrical Engineering, University of West Bohemia, 30100 Pilsen, Czech Republic; marsorm@students.zcu.cz

**Keywords:** Ti6Al4V, laser machining, wettability, biocompatibility, dental implants, surface microstructure

## Abstract

This article explores the enhancement of material surface properties of Ti6Al4V, potentially applicable to dental implants, through ultra-short pulse laser systems. This study investigates potential connections between surface wettability and biocompatibility, addressing the challenge of improving variability in material properties with specific laser treatment. Several designed microstructures were manufactured using a picosecond laser system. After that, the wettability of these structures was measured using the sessile drop method. The basic behavior and growth activity of biological cells (MG-63 cell line) on treated surfaces were also analyzed. While the conducted tests did not conclusively establish correlations between wettability and biocompatibility, the results indicated that laser treatment of Ti6Al4V could effectively enlarge the active surface to better biological cell colonization and adhesion and provide a focused moving orientation. This outcome suggests the potential application of laser treatment in producing special dental implants to mitigate the issues during and following implantation.

## 1. Introduction

Modern dentistry almost cannot be imagined without dental implantology. For successful implementation of implants, the implant itself with its specific properties and the implant acceptor, i.e., a living organism with all its properties (genetic and epigenetic), play a crucial part [1,2,3]. Each type of implant can be characterized by its biocompatibility. Biocompatibility refers to the ability of substances to interact with the biological environment harmoniously. Their key feature is the ability to minimize toxic emissions, not irritate tissues, not induce carcinogenicity, and resist corrosion and wear. These materials are often regarded as optimal for promoting cell growth and tissue integration [4]. It is necessary to describe the basic reaction between live cells and the material of implants. This is very simple: either the cell is rejected by the material and the life of the cell ends, or the bioactive cell is accepted by the material, in which case a population of cells colonizes the free surface area. However, the step from rejection to acceptance in the case of so-called biocompatible materials is very wide, because it depends on the material’s chemical composition, energy, roughness and topography (each acting separately or synergistically) [4,5]. Thus, it can be assumed that a biological cell’s ability to adhere to the surface will depend on its ability to make some form of contact with the substrate; in the simplest form, this could be focal contact or some form of other adhesion [6]. However, this may mean that the formation of adhesive zones depends on the interaction of the cell surface with another surface. Because this is a very dynamic process, it is easier to focus only on one indicator, which is the mobility of the cell on the surface of the substrate. If the surface is altered in some way (for example, if specific macroporous structures are formed on it) [7], it can be assumed that the formation and dynamics of the cell’s adhesive properties will be affected. Peri-implantitis, an inflammatory condition disturbing the soft tissue surrounding the implant interface, plays an essential role in causing implant failure [8].

Plasma spraying, laser ablation, sandblasting, or electron beam structuring are technologies used to modify the surface properties [9,10,11]. As for the dental implants themselves, their surfaces are commercially treated by several methods besides laser ablation: etching, electrochemical anodization processes, blasting and etching, hydroxyapatite coating, and plasma spraying [12].

Laser micromachining represents a useful tool for manufacturing specific microstructures with various shapes on biocompatible materials [13,14]. Using ultrashort pulse lasers, various shapes of microstructures can be produced. One of the benefits of laser micromachining is the possibility of machining various materials without the occurrence of residual heat in the machined material [15]. However, shape and dimension specifications are crucial for the biocompatible properties of implants, such as dental implants.

Available scientific articles focus on finding suitable parameters for the preparation of highly biocompatible surfaces. The main subject is still a common phenomenon called osseointegration, during which the healing of the dental implant into the bone occurs. A high level of osseointegration can be associated with a strong connection between bone and dental implants. This phenomenon is caused by the integration of cells on the surface of the implant and the cultivation of culture, which is connected to the material and bone. These types of connections can have different levels of stability, mechanical load capacity, and lifetime [1]. Different types of surface microstructures benefit the adhesion and growth of the biological cells on the surface, especially the structures of grooves. Martínez et al. [16] reported that the optimal depth and width of the grooves depend on the type of cell selected for the cultivation. The examined widths of the grooves were from 200 nm to 10 µm, and the depths were from 300 nm up to 5 µm. The authors describe how these microstructures can also affect the direction of adherence of the biological cells. The motivation for having specific oriented and placed cells is to form a cell culture where the cells must have a specific position to ensure they can proliferate and communicate. It was also discussed that surface biocompatibility is influenced not only by the dimension of the surface microstructures but also by the surface’s wettability.

Wettability is a material property that reduces surface tension and allows a liquid to spread across its surface [17]. Whether the surface is wetted with liquid or not is determined by the size of the contact angle (CA/Θ) between liquid and surface. The contact angle can be determined by direct (goniometric) or indirect (tensiometric) methods of measurement [18]. Using the parameter of the contact angle, the surfaces can be divided into four main groups, which are hydrophilic (CA < 90°), neutral (CA = 90°), hydrophobic (CA > 90°), and superhydrophobic (CA > 150°) [19]. Amongst the superhydrophobic surface belongs, for example, the surface of lotus leaf, which can be higher than 160° C [20]. Hydrophilic surfaces (CA < 90°) and surfaces with higher roughness enhance osteoblast proliferation, which can lead to better tendencies to osseointegrate [8,21]. As the wettability is well discussed, it is possible to find several academic publications that focus on the production of surface microstructures that are changing the wettability of the treated surface using ultrashort pulse laser systems [22,23,24,25,26,27,28,29].

There are relatively few scientific articles that focus on the connection between surface wettability and biocompatibility of the materials; therefore, this topic is explored in this publication. In particular, the effect of laser machining of Ti6Al4V material on the behavior of biological cells will be investigated.

## 2. Materials and Methods

### 2.1. Materials

**Figure 1:**
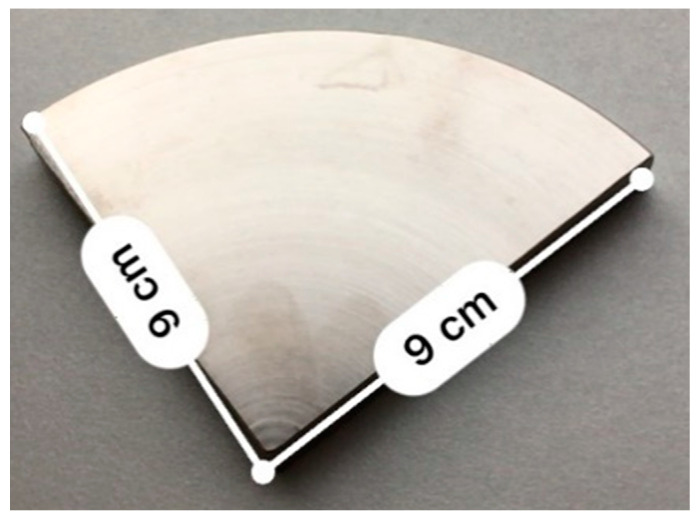
Ti6Al4V sample.

The biocompatible material Ti6Al4V was used for the testing of the influence of laser treatment of material on the behavior of biological cells. This alloy was chosen because of its availability on the market and its high popularity in the implant industry; also, this type of material has specific mechanical and chemical properties such as excellent corrosion resistance or high specific strength, which can be beneficial for biomedical metallic implants. The range of use of Ti6Al4V alloys in the implant industry can be increased by adding passive layers or biocompatible stabilizers [30]. For the experiment, a sample of the material was purchased in the shape of a rod, which was divided by a conventional turning machine and then cut into smaller parts using a bandsaw. The shape of these samples was a quarter of a cylinder with a radius of 90 mm and a height of 20 mm (Figure 1). Also, the roughness of the samples was measured using the Mitutoyo SURFTEST SJ-301 (Mitutoyo, Sakado, Japan) device, and the average value of Ra roughness of the samples was measured as 0.38 µm.

### 2.2. Laser System

For the experimental examination, the picosecond pulse Nd:YAG laser was used. Specifications of this laser system are listed in Table 1.

This laser system works on the principle of direct laser ablation (DLA), a non-contact versatile structuring technology that can be applied to fabricating a wide range of surface elements [31]. Two configurations can be used when working with DLA technology. Both configurations use a laser beam expander to adjust the size of the laser diameter spot size subsequently. The first configuration operates using focusing optics that are placed on the vertical axis. The transmission of the laser beam to the sample surface is enabled by the movement of the sample itself in the XY axis. The second one uses a scanning head (e.g., a Galvo scanner (ScanLab, München, Germany) that moves in the plane of the XY by the laser beam on a stationary sample. This second type was used in the experimental part of this article. One of the disadvantages of DLA technology is relatively low productivity (compared, for example, to DLIP technology [32]), which is limited because it uses only one focused laser beam. However, its potential lies in its wide versatility.

**Figure 2:**
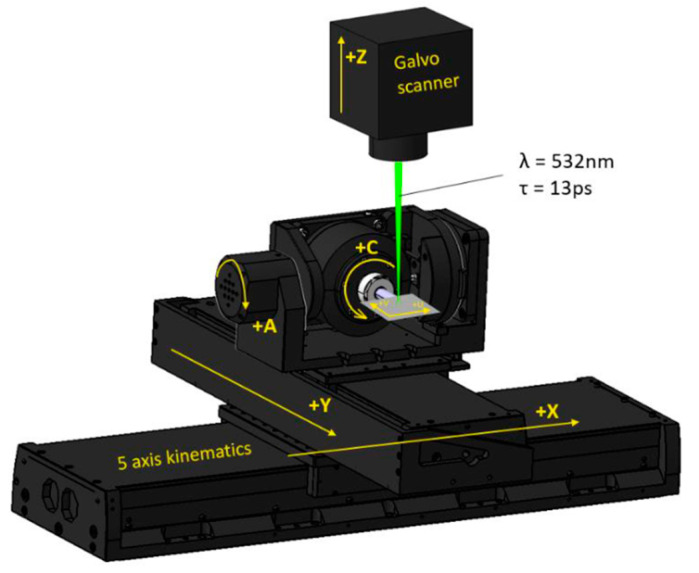
Scheme of the laser kinematics [33].

The specific positions of the machined Ti6Al4V samples were determined using multi-axis stages (Aerotech, Pittsburgh, PA, USA). The representation of the configuration of the stages A, C, X, Y, Z) and optical axes of the galvo-scanner is shown in Figure 2.

### 2.3. Surface Microstructures and Processing Parameters

Considering available scientific literature and previous experience in the field of laser surface structuring and in addition to minimizing the time needed for evaluation experiments, four types of surface microstructures were designed (Line, Line + LIPSS, Cross, Platex) whose specific shapes should lead to changes in the wettability and bioactivity of treated surfaces. The shapes and dimensions of these microstructures are listed below.

- Line

**Figure 3:**
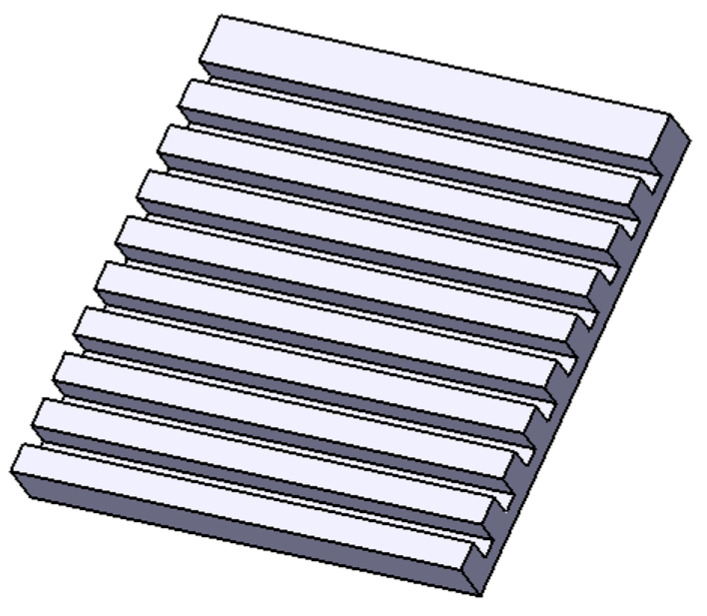
Model of surface microstructure Line.

This microstructure consists of parallel grooves with a width of 15 µm, a pitch of 20 µm, and a depth of 10 µm (Figure 3).

Microstructure Line was made in two modifications (Line and Line + LIPSS) with different setups of the laser system. The modifications differed in the use of linear or circular polarization. Different setups of polarization can be used to control a phenomenon called laser-induced periodic surface structures (LIPSS). LIPSS is a phenomenon observed when a material is treated with a high-intensity polarized laser—typically with picosecond or femtosecond pulse lengths. The changing of the polarization angle can be utilized to control the direction of the LIPSS [34]. This process spontaneously generates a repeating pattern of structures (their size is in the order of nanometers) on the material’s surface, altering its optical properties and enabling the formation of holograms. Additionally, LIPSS can create unique features that can lead to uneven hydrophobicity, which can be used to control liquid flow on the surface [35].

For the first modification of the surface microstructure line, circular polarization was used to suppress the formation of the LIPSS. For the second modification (Line + LIPSS), linear polarization was used, which resulted in the spontaneous generation of nanoscopic structures with specific orientation on the bottom of the fabricated grooves, contrary to the structure Line.

- Cross

**Figure 4:**
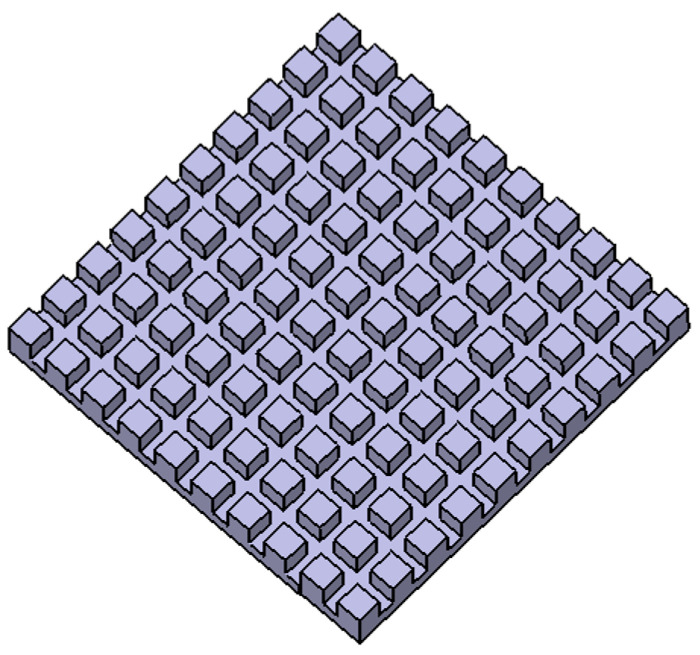
Model of surface microstructure Cross.

This microstructure consists of parallel grooves and also grooves which are shifted by 90° with a width of 15 µm, a pitch of 20 µm and a depth of 23 µm (Figure 4).

- Platex

**Figure 5:**
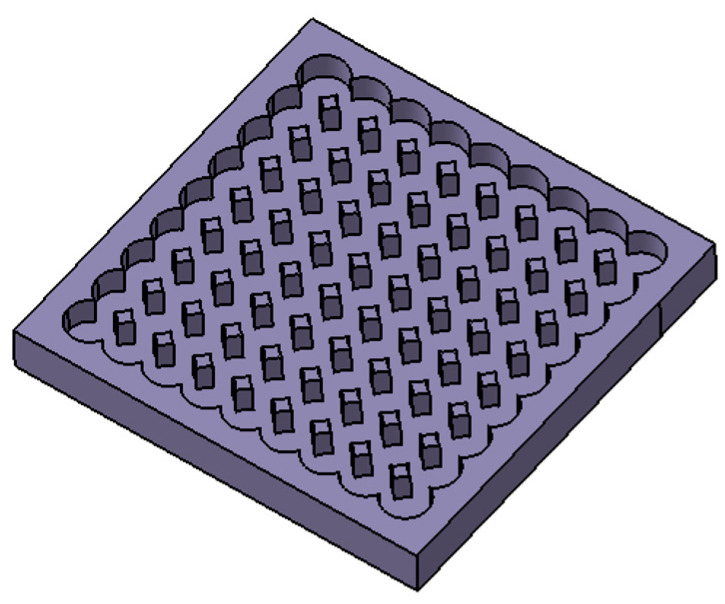
Model of surface microstructure Platex.

This microstructure consists of holes that are arranged in the grid with a radius of 8 µm, a pitch of 11 µm, and a depth of 15 µm (Figure 5).

The described picosecond laser system was used to produce the designed surface microstructures.

For the production of each designed surface microstructure exact laser processing parameters were set. The values of these processing parameters are shown in Table 2.

The microstructures were fabricated in the shape of squares whose dimensions were 7 × 7 mm. After fabrication, each surface microstructure was washed in an ultrasonic cleaner with pure isopropyl alcohol and then measured using an Olympus Lext (software version 2.1.2.8087) confocal microscope (Olympus, Tokyo, Japan) to verify the fabrication accuracy and compliance with each structure’s designed dimensions.

### 2.4. Evaluation Methods

Two methods were used to evaluate the fabricated samples. The first one was the so-called sessile drop method, which is the standard method for determining wettability. In this technique, a precise volume of distilled water, typically ranging from 1 to 10 μL, is carefully dispensed onto the sample surface using a specialized syringe [36]. The interface between the water droplet and the surface was then recorded in real-time using a video camera. A software program was employed to measure the contact angle formed by the droplet, which served as an indicator of surface-wetting behavior.

The second method used to determine the biocompatibility of the surface structures (in vitro) consisted of several steps. Firstly, it was necessary to cultivate the cell culture before using it. The cultivation procedure before testing consists of incubation of the cells in a CO_2_ thermostat set to a 5% CO_2_/air mixture at 37 °C and 95% relative humidity. This environment served as the mother culture from which the necessary number of cells for experimental cultivation was harvested using conventional trypsinization techniques. Then, the experimental cultivation involves growing cells on the sample surface, typically for three days or approximately three consecutive cell division cycles. Following this incubation period, the cell culture is halted, and the cells are treated with fixation and coloring techniques. The area covered by the cells is subsequently quantified or determined. The SEM method was used to determine the result. As a cell culture, the MG63 (human osteosarcoma) was chosen, and the cultivation medium was EMEM medium with 10% fetal bovine serum, antibiotics, antimycotics, and glutamine.

To elucidate the inherent variability present in the obtained results, it is necessary to determine the standard deviation (SD). This statistical parameter quantifies the extent to which values are distributed around the mean and is calculated as the square root of the variance [37].

## 3. Results

All four designed microstructures were first fabricated using a picosecond ultrashort pulse laser system with specific setup and processing parameters (see Section 2.3). After fabrication, each surface microstructure was washed in an ultrasonic cleaner with pure isopropyl alcohol and then measured using an Olympus Lext confocal microscope (Olympus, Tokyo, Japan) to verify the fabrication accuracy and compliance with each structure’s designed dimensions.

**Figure 6:**
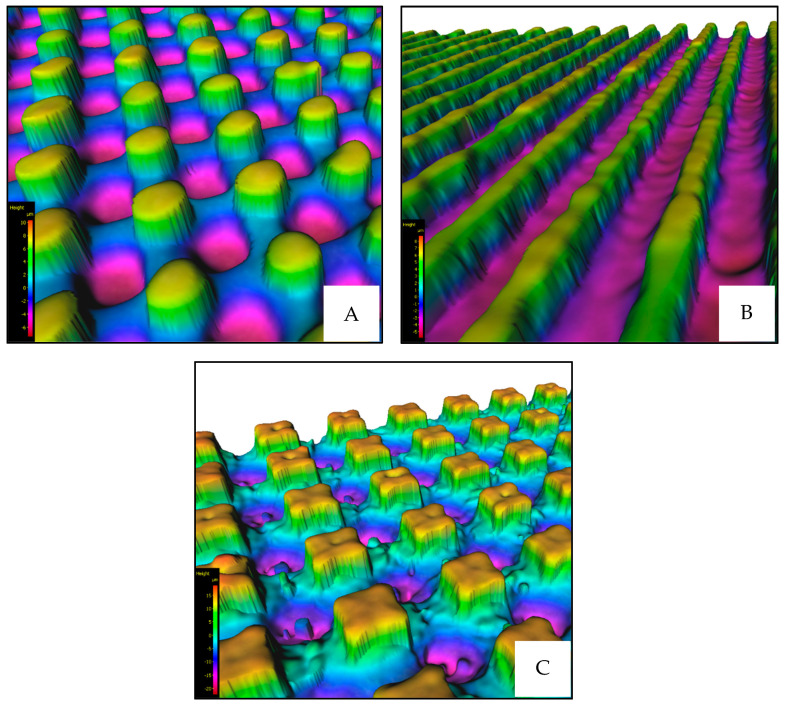
Examples of fabricated microstructures ((**A**)—Platex, (**B**)—Line, (**C**)—Cross).

Based on these measurements, it was verified that the dimensions of the individual microstructures corresponded to the designed dimensions (Figure 6).

**Figure 7:**
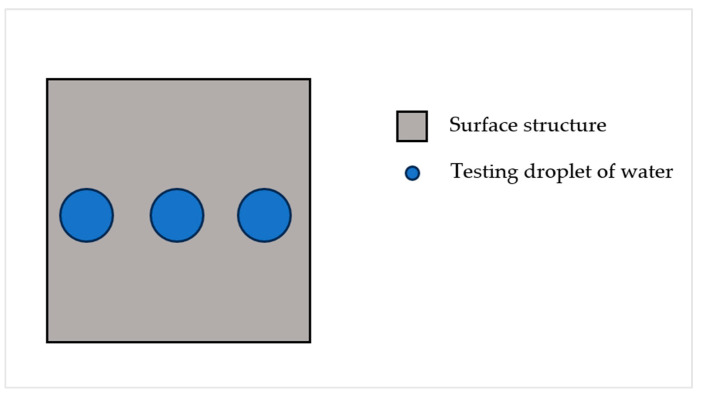
Schematic overview demonstrating the methodology used in determining wettability.

After that, the evaluation of the wettability was processed. For this measurement, the sessile drop method was used (Section 2.4). Distilled water was chosen as a medium, and this whole process of measurement of wettability was performed using a Krüss DSA30E (Krüss, Hamburg, Germany) device. Utilizing the device’s programmable features, measurements were automated for optimal precision in repetition. This entailed depositing three consecutive drops onto each 7 × 7 mm area (each surface microstructure), with each drop (volume of 5 µL) precisely measured for 30 s and repeated exactly 30 times (Figure 7).

**Figure 8:**
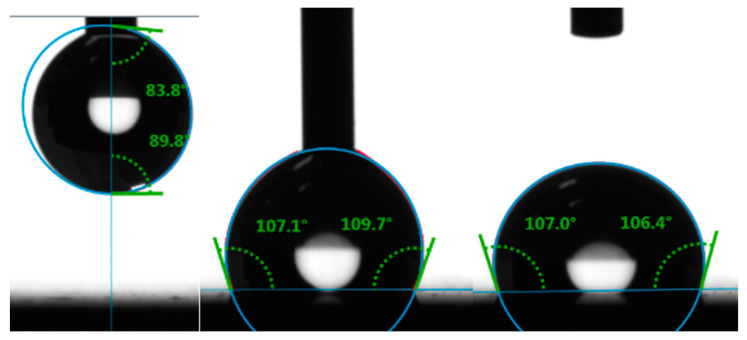
Deposition process during sessile drop wettability measurement.

Additionally, as illustrated in Figure 8, droplet angles were measured from both sides to capture left and right angles.

Subsequently, mean values and overall averages were calculated (Table 3).

The wettability testing showed that the laser fabrication of surface microstructures on the surface of the test samples resulted in a change in the wettability properties. It can also be observed that the production of different microstructures (different shapes and dimensions) leads to different values of CA. The most hydrophobic surface was the surface with the microstructure of Lines (CA = 85.32°), and the most hydrophilic microstructure was the microstructure of Platex (CA = 11.18°).

**Figure 9:**
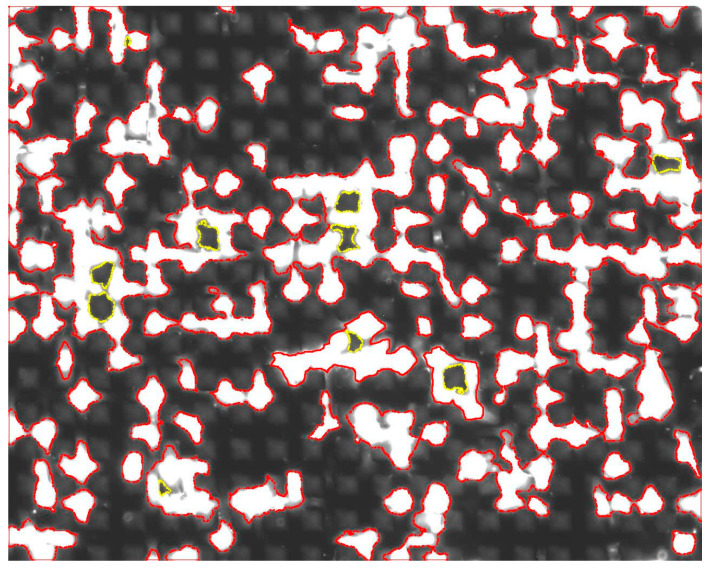
Image from picture analysis.

The last set of tests focused on the evaluation of biocompatibility of surface structures. The adherence, locomotion, and colonization of the biological cells were examined in particular. For this type of testing, the cell line MG63 was used in three types of generation, indicating that the optical evaluation of the percentual coverage of the surface with biological cells was completed after 24, 48, and 72 h (Section 2.4). Regarding the evaluation, a total of 12 samples were tested (3 samples of each surface structure). The measurements of one sample involved capturing images at 16 distinct locations using a linear electron microscope (SEM). These images were subsequently subjected to image analysis (Figure 9).

**Figure 10:**
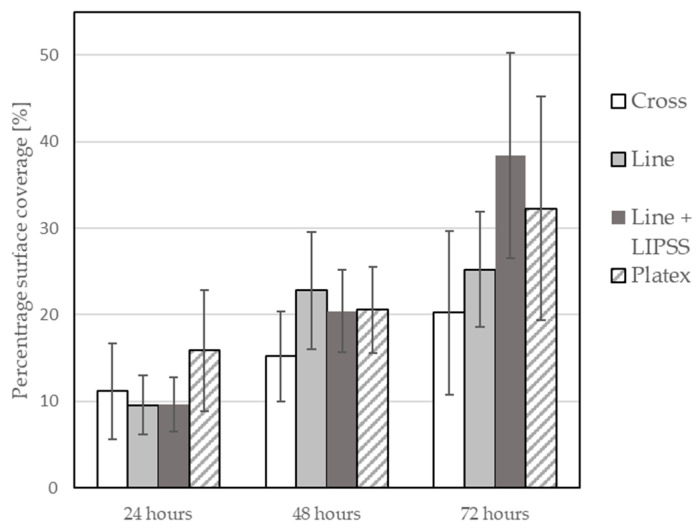
Graph of biological cell colonization.

Within the scope of this analysis, the percentage of image coverage by biological cells was calculated. Picture analysis of the size of the area covered by the cells was performed by adding the size of the areas bordered in red and subtracting the size of the areas bordered in yellow from the resulting size. Subsequently, the maximum, minimum, and mean values of percentage surface coverage were calculated for each image, and then the average value of mean values across all three tested samples was calculated. This measurement was repeated after 24, 48, and 72 h of cell growth. The average of the mean values of the percentage surface coverage by biological cells for all surface structures were plotted in a graph with the measurement’s standard deviation (Figure 10).

The percentage surface coverage by biological cells was also tested on the samples without laser treatment. In this measurement, six samples were measured only after 72 h of growth. The resulting average value of mean values of percentage surface coverage for laser untreated samples was 21.48%. This result suggests that using a picosecond laser system for the treatment of the Ti6Al4V surface can lead to potential changes in the view of biological cell colonization, especially the manufacture of Line + LIPSS surface structure, and it can be noted that some surface structures have a bigger impact than others.

**Figure 11:**
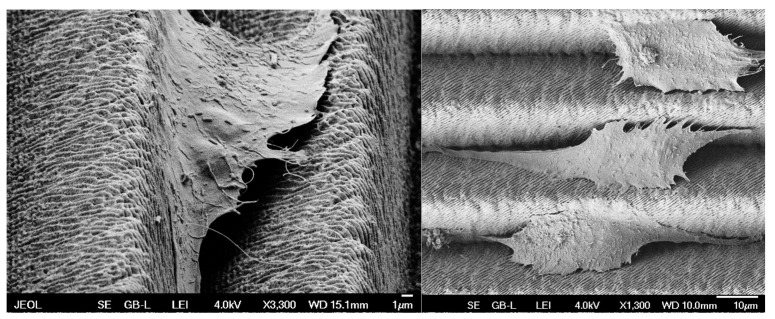
Cell adherence to surface microstructures.

Based on these tests (wettability and biological cell colonization), it can be observed that a strong correlation between wettability and cell colonization has not been fully established (Figure 8 and Figure 10). It can be observed that over time, there is an increase in the number of biological cells on the test samples; it can also be noted that the production of different types of surface structures will affect the cell growth rate differently; however, there is not much difference between the most hydrophobic structure and the most hydrophilic structure in terms of biological cell coverage. However, it should be noted that the analysis of the acquired images had its limitations, which consisted of the impossibility of measuring the area under the cell that adhered to the top of the formed structures (Figure 11). So, it was not clear how many cells could be lying on top of each other.

**Figure 12:**
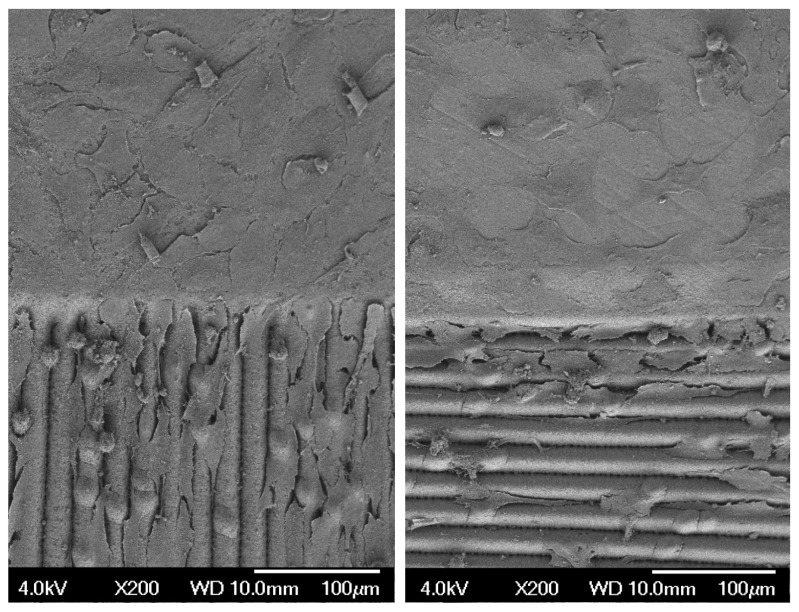
Locomotion of cell depending on the orientation of surface microstructures.

Nevertheless, new insights have been revealed, particularly in the relationship between cells and the morphology of microstructures. It was observed that cells generally tend to take up positions within grooves (Figure 12) and, in some cases, even lie on top of each other so that they overlap. From this point of view, the influence of the fabricated microstructure can be seen as a differentiating factor since the resulting active area that the cells can occupy is higher. At the same time, the cells are targeted to be oriented into the grooves.

## 4. Discussion

The main idea of this article was to investigate the behavior of biological cells on titanium alloy (Ti6Al4V), which was treated by an ultrashort pulse laser. Several publications suggest that laser surface treatment of biocompatible materials can lead to improvements in their mechanical and physical properties. The use of these materials with special properties can play a key role in the field of medical implants.

In this study, several experiments were undertaken. For these experiments, a picosecond pulse Nd:YAG laser was used. Firstly, four different surface microstructures were designed with detailed dimensions and shapes for the creation of surface structures that influence the wettability of the surface because there is a view, according to available studies, that there is a link between wettability and surface bioactivity. Several tests were conducted to set up ideal laser parameters for the ideal fabrication of designed surface microstructures. These parameters were laser power, frequency, repetition, and overlap of laser pulses. The correctness of the fabrication of designed surface microstructures was verified by using the optical microscope Olympus Lext. The dimensions of all four designed surface microstructures were fabricated with sufficient accuracy.

Regarding wettability testing, it was observed that the creation of surface microstructures leads to a change in surface wettability. The biggest CA was measured on the surface microstructure of Lines (85.32°), and the smallest was on the microstructure of Platex (11.18°). This indicated that with the defined shapes and dimensions of the structures, it is possible to control the surface wettability of the treated surface in a targeted manner.

This was followed by the measurement of bioactivity on the treated surfaces and its colonization with the cells of the MG63 cell line. Unfortunately, a strong correlation between wettability and biocompatibility of the surface was not observed. This may have been due to the fact that surface wettability is only one of many surface characteristics that can affect the behavior of the bioactive cells, such as the chemical properties of surfaces. Verification of this hypothesis may be the aim of further work. However, the results of the colonization of these surfaces revealed several new phenomena. First, the trend in per cent coverage did not consistently increase over the time of cell generation (one generation corresponds to a 24-h cycle). For example, this phenomenon can be explained by the cells triggering a mechanism that accelerates the whole process in the first hours of colonization at the expense of later activity. Another interesting phenomenon was that the grooves leading from the polished surface guided the individual cells within the grooves. The difference occurred at points along the grooves, where the cells found it very difficult to cross the grooves in a direction perpendicular to the longitudinal axis of the grooves, and their number was significantly reduced with each successive groove.

## 5. Conclusions

In the course of this comprehensive study, significant observations emerged regarding the effects of laser treatment on surface properties. It was observed that specific surface microstructures can be used in the fabrication of different medical implants, such as dental implants. Using this knowledge, it is possible to manufacture such implants with specific areas that can have different properties regarding wettability, cell colonization, or even cell locomotion. This specification of the implant could increase the chances of tissue acceptance and possibly even prolong its lifespan. To conclusively verify this assumption, conducting additional tests is essential. These tests should examine, for example, different cell lines and explore the fabrication of these structures on flat surfaces and across varied topographies.

## Figures and Tables

**Table 1:** Laser source and optical path specification.

Laser System	Data
Laser source	Atlantic, Ekspla (Ekspla, Vilnius, Lithuania)
Galvo-scanner	IntelliScan, ScanLab (ScanLab, München, Germany)
Wavelength	532 nm
Maximum pulse energy	60 µJ
Pulse repetition rate	0.2–1 MHz
Duration of laser pulse	13 ps
Laser beam spot	~25 µm
Focal length	160 mm
Beam quality	M^2^ ≤ 1.3
Polarization	lineal/circular

**Table 2:** Processing parameters used to create designed microstructures.

Surface Microstructure	Power [W]	Frequency [kHz]	Pulse Energy [µJ]	Number of Repetitions	Polarization
Line	800	500	1.6	140	circular
Line + LIPSS	800	500	1.6	140	linear
Cross	800	500	1.6	290	circular
Platex	500	200	2.5	800	circular

**Table 3:** Values of CA for all surface microstructures.

Surface Microstructure	Average from Mean Values CA [°]	Difference of CA from Reference Surface [°]
Lines	85.32 (±23.9)	+6.45
Lines + LIPSS	38.95 (±22.2)	−39.92
Cross	15.44 (±2.94)	−63.43
Platex	11.18 (±1.79)	−67.6

## Data Availability

Data are contained within the article.
